# Discriminating Grotesque from Typical Faces: Evidence from the Thatcher Illusion

**DOI:** 10.1371/journal.pone.0023340

**Published:** 2011-08-31

**Authors:** Nick Donnelly, Nicole R. Zürcher, Katherine Cornes, Josh Snyder, Paulami Naik, Julie Hadwin, Nouchine Hadjikhani

**Affiliations:** 1 School of Psychology, University of Southampton, Southampton, United Kingdom; 2 Brain Mind Institute, Swiss Federal Institute of Technology, Zurich, Switzerland; 3 Department of Radiology, Harvard University, Cambridge, Massachusetts, United States of America; Beijing Normal University, China

## Abstract

The discrimination of thatcherized faces from typical faces was explored in two simultaneous alternative forced choice tasks. Reaction times (RTs) and errors were measured in a behavioural task. Brain activation was measured in an equivalent fMRI task. In both tasks, participants were tested with upright and inverted faces. Participants were also tested on churches in the behavioural task. The behavioural task confirmed the face specificity of the illusion (by comparing inversion effects for faces against churches) but also demonstrated that the discrimination was primarily, although not exclusively, driven by attending to eyes. The fMRI task showed that, relative to inverted faces, upright grotesque faces are discriminated via activation of a network of emotion/social evaluation processing areas. On the other hand, discrimination of inverted thatcherized faces was associated with increased activation of brain areas that are typically involved in perceptual processing of faces.

## Introduction

**Figure 1:**
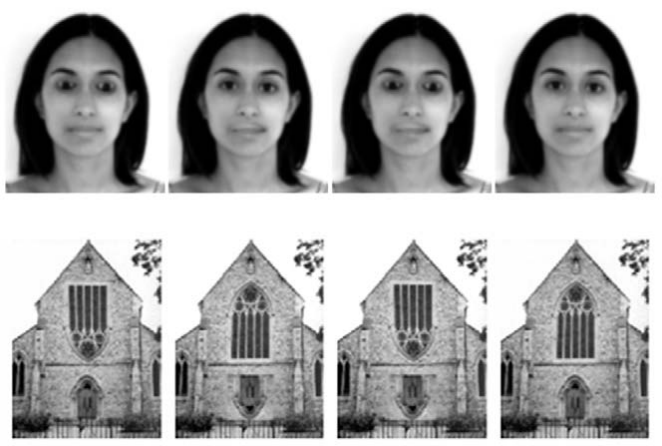
Examples of the faces and churches used in the behavioural and imaging tasks. Note the example of the face image is not one of the stimuli used in the experiment, but an illustrative example of the Thatcher illusion as instantiated in this study.

The Thatcher illusion (see Figure 1) is formed by inverting eyes and mouths in otherwise upright faces [1]. Faces are perceived as typical when inverted but grotesque when presented upright. Recent explanations of the illusion have given a central role to configural processing [2], [3]. The present paper explores this issue.

Face perception is usually considered in terms of activation in a network of brain areas involved in face identification (fusiform face area (FFA), inferior occipital gyrus (IOG)), and in emotional/social evaluation (e.g. amygdala (AMY), superior parietal lobule (SPL), inferior parietal lobule (IPL) and inferior frontal cortex (IFC) [4], [5]. In addition, emotional/social evaluation in general is associated with medial prefrontal and posterior cingulate activation [6].

Previous exploration of brain responses to thatcherized faces has been restricted to a set of event related potential (ERP) studies focussing on occipital-temporal brain regions. These studies explored the effect of thatcherized versus typical faces on the amplitude and latency of the N170 and other markers of early face processing. For example, Boutsen, Humphreys, Praamstra and Warbrick [7], showed evidence of delayed N170 with reduced amplitudes for thatcherized faces versus typical faces when participants performed an oddball task: these results holding for both upright and inverted stimulus presentations although all latencies were longer for inverted than upright faces. However, other studies requiring different judgements such as an identity or gender decision [8], [9] have reported increased amplitude of N170 to thatcherized faces relative to typical faces. What is apparent is that thatcherized faces elicit different (in terms of both amplitude and latency) ERP responses from typical faces, although the nature of this difference is dependent on task. Nevertheless, with one single exception [10], we know nothing of the responses of other brain regions when discriminating typical from thatcherized faces.

In the present study, we explore brain regions associated with the discrimination of thatcherized from typical faces. Using a two alternative forced choice (2AFC) simultaneous presentation, participants were required to discriminate thatcherized faces from matched typical versions when stimuli are both upright and inverted [11], [12]. This simultaneous 2AFC paradigm reveals the areas active when making decisions about which ones of pairs of faces are the most grotesque.

We also report a behavioural study. This study develops the basic 2AFC discrimination task by manipulating the number of features differing between face pairs (just eyes, just mouths or both eyes and mouths) and the impact of cuing (by either cuing or not) to critical features. By exploring the role of both the number of feature changes and cuing on discrimination, as well as orientation, we demonstrate the pre-eminent role that eyes play in discriminating thatcherized from typical faces. In this behavioural study we also report on the same manipulations but conducted on church stimuli, where windows and doors replace eyes and mouths (see figure 1). This was done to confirm that the 2AFC discrimination task also demonstrates the face specificity of the illusion.

The results of the behavioural study confirm that faces are much more affected by inversion than churches [13] and dominance for responding to eyes over mouths with unfamiliar faces [14]. The neuroimaging task demonstrates that the medial prefrontal cortex (mFC) and subcallosal cortex (SubCal) are active when discriminating upright thatcherized and typical faces, especially when attention is cued to eyes. In contrast, discriminating between inverted faces relies on activation of extended face processing areas associated with the FFA.

## Results

### Behavioural task

First we explored the face specificity of the illusion by analyzing mean RT and error rates across all faces and churches, using a 2 (Stimulus: Faces versus Churches)×2 (Orientation: Upright versus Inverted) ANOVA repeated over both factors. This demonstrated a significant interaction between Stimulus and Orientation (*F*(1,16) = 20.20 and 43.61 *p*<0.01, for RTs and errors respectively). Inversion affected faces more than churches. Discriminations of faces and churches were matched on RT and accuracy in upright faces/churches but not when inverted. The simultaneous 2AFC Thatcher discrimination task does demonstrate the face specificity of the Thatcher illusion.

**Figure 2:**
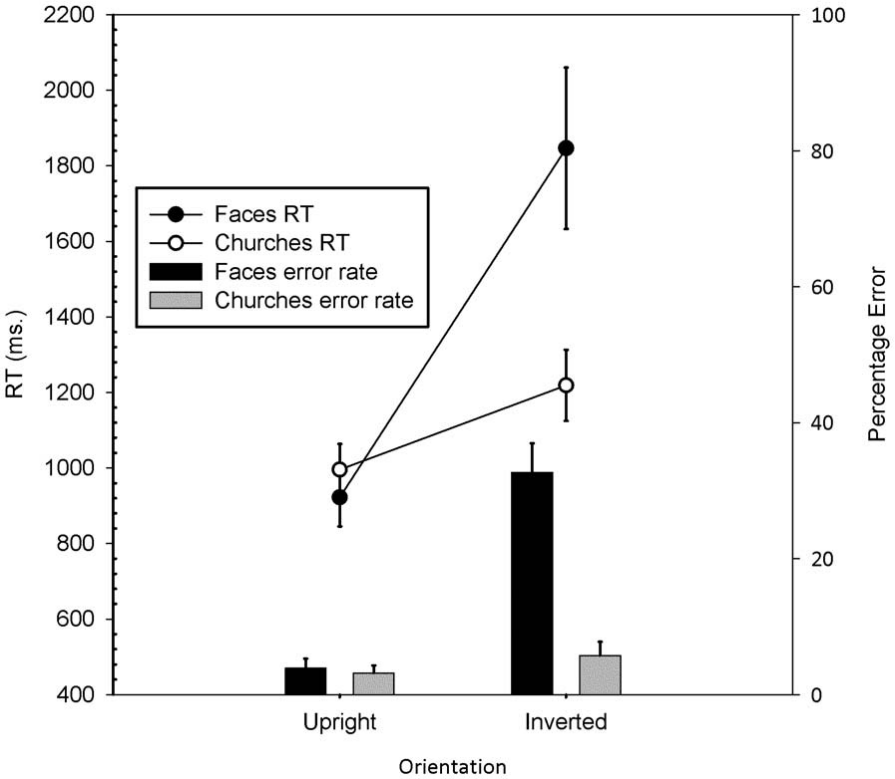
RTs and error rates (with standard errors) for faces and churches in the behavioural task: aggregated across conditions.

**Figure 3:**
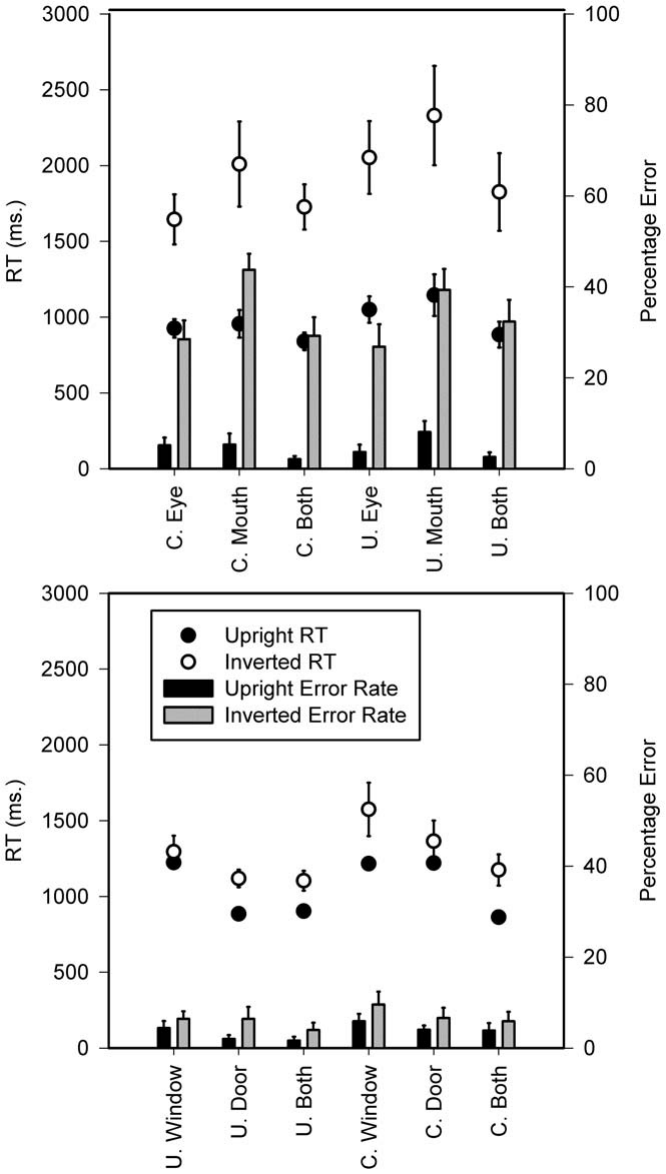
RTs and error rates (with standard errors) for faces (left panel) and churches (right panel) in the behavioural task.

Second we explored how both the number of features manipulated and cuing influenced discrimination of Thatcher from typical faces. RTs and error rates were analysed separately in a 3 (Trial type: Eye versus Mouth versus Two features (eyes and mouth)×2 (Orientation of context: Upright versus Inverted)×2 (Cue: Cued versus Uncued) repeated measures ANOVA (see Figures 2 and 3). Note that the feature data were formed by averaging across all trials in which both features had been modified (blocks 2 & 4 of eye condition and blocks 2 & 4 of mouth condition): prior analyses having revealed no difference across two-feature conditions for RTs or errors (*F*<1 and *F*(1,16) = 2.87 respectively; see Figure 3). Crucially in the RT analysis, the main effects of Trial type, Orientation and Cuing were all significant (*F*(2,32) = 77.31, *F*(1,16) = 31.40 and *F*(1,16) = 57.18 respectively, all *p*<0.01). The interactions between Trial type and Orientation (*F*(2,32) = 7.81, *p*<0.01), Trial type and Cue (*F*(2,32) = 69.17, *p*<0.01), Cue and Orientation (*F*(1,16) = 18.26, *p*<0.01) and Trial type, Cue and Orientation (*F*(2,32) = 9.95, *p*<0.01) were all significant. Responses in the two-feature conditions, where both eyes and mouths were manipulated, were always faster than in the single-feature conditions, where only eyes or mouths were manipulated. Only when faces were inverted and participants were cued to the eyes was this not the case. In this case, RTs were not statistically different. With respect to error rates, the main effects of trial type and orientation were significant (*F*(2,32) = 7.87, *p*<.01; *F*(1,16) = 79.80, *p*<.05 respectively). Responses were more accurate for upright than inverted faces. Single-feature mouth trials were responded to less accurately than all single-feature eye trials and two-feature trials. The main effect of cue was not significant (*F*(1,16) = 1.01). In addition, all interactions failed to reach significance (all *F* ratios <2.32). We conclude from these data that the 2AFC Thatcher task demonstrates the classic findings of face specificity [13] and feature dominance for eyes over mouths [14].

### Imaging task

**Figure 4:**
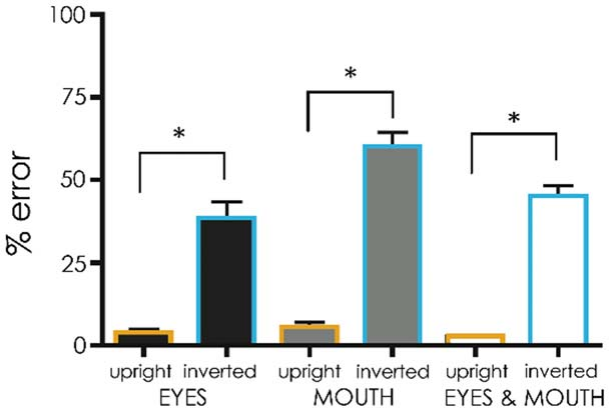
Percentage error rates (with standard errors) for the fMRI task.

The imaging task was a reduced version of the behavioural task where all trials were cued and only faces were shown. The error rates from this task were analysed in a 3 (Trial type: Eye versus Mouth versus Two features)×2 (Orientation of context: Upright versus Inverted) repeated measures ANOVA to confirm the presence of the Thatcher illusion for participants in the imaging study. Importantly, the main effect of Orientation was significant (*F*(1,15) = 225.00, *p*<0.001; see Figure 4). In addition, the interaction between Trial type and Orientation was also significant (*F*(2,30) = 9.53, *p*<0.001). This was due to a higher error rate on mouth only trials than both other trial types for inverted but not upright trials.

### Whole brain analysis

When modifications were made to the eyes or to the mouth, discriminating upright thatcherized from typical faces elicited more activation in the mFC/SubCal, in the posterior cingulate (Pci)/precuneus (PreCun) cortex, in the superior frontal gyrus as well as in the middle temporal and the parahippocampal gyrus (*p*<0.05 after FDR correction). In addition, increased activation for upright faces in the left postcentral gyrus was only found in the mouth-cued condition.

**Figure 5:**
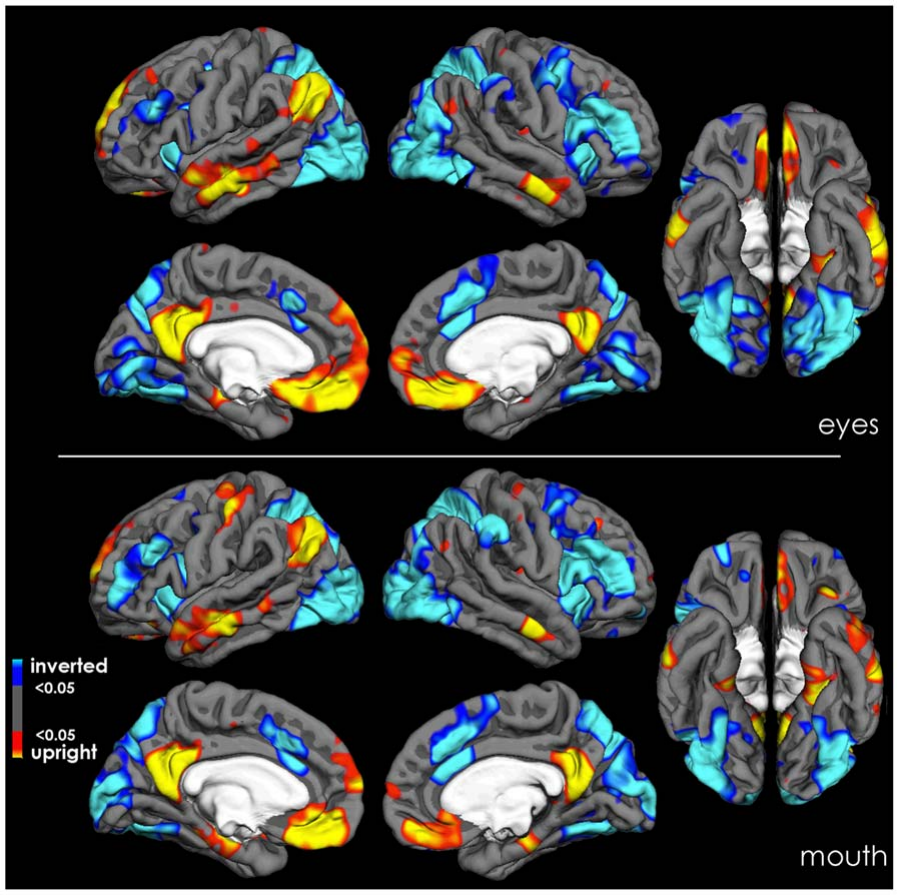
Areas of significant activations in the analysis of upright vs. inverted faces for the eye-cued (upper panel) and mouth-cued (lower panel) conditions.

The discrimination of features presented in inverted faces elicited in general more activation than discrimination in upright faces. Areas of increased activation were found in the inferior frontal cortex, the middle frontal gyrus, the middle cingulate cortex, the superior and inferior parietal cortex, the lateral occipital cortex, the inferior occipital cortex and the lateral and medial fusiform gyrus (*p*<0.05 after FDR; see Figure 5).

### *A priori* ROI analysis

**Figure 6:**
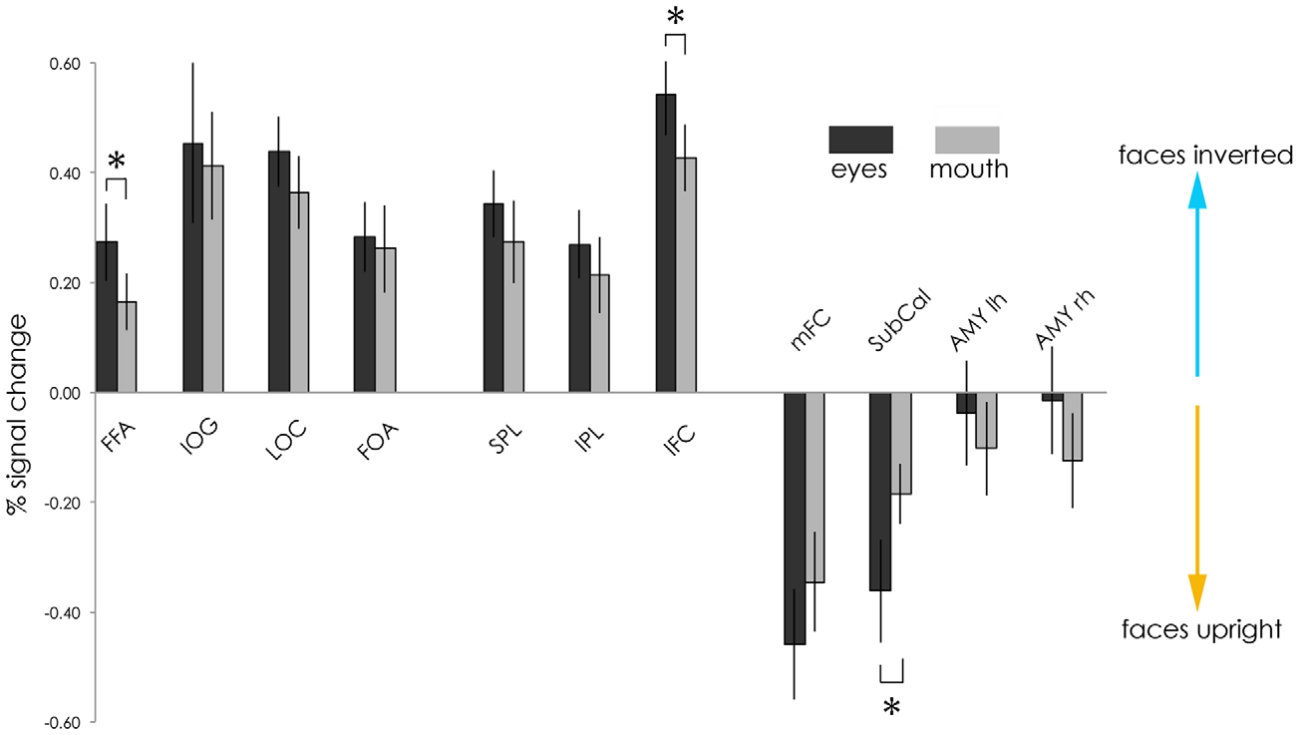
Percent BOLD signal change (with standard errors) in ROIs for the contrast of upright versus inverted faces for single feature conditions.

The activation between the contrast of upright and inverted faces was determined with a *t*-test against zero. Upright faces led to significantly increased activation in the mFC and the SubCal in both the eye and mouth conditions (*p*<0.01; see Figure 6).

Inverted faces on the other hand, whether they were cued to eyes or mouths, activated areas from the face-processing network including the FFA, the IOG as well as SPL, IPL and the IFC (all *p*<0.01). The *a priori* ROIs that are involved in object perception, the FOA and the LOC were also significantly more activated for inverted than for upright faces in both eye and mouth conditions (all *p*<0.01). The amygdala activation was not significant for either condition.

Comparing the eye and mouth conditions, there was significantly increased activation in the eye condition in the SubCal (*t*(17) = 2.04, *p*<0.05) for upright faces, and significantly increased activation in the eye condition in the IFG (*t*(17) = −2.03, *p*<0.05) and FFA (*t*(17) = −2.10, *p*<0.05) for inverted faces.

### Correlations between ROIs

Functional connectivity between the right FFA and the other face-processing and emotion/social-processing cortical areas of the right hemisphere and both amygdalae were assessed from time courses of responses to thatcherized and typical faces. Time-courses were extracted from all voxels within the right FFA and all other selected ROIs. For each ROI they were averaged across blocks within each condition. Two-tailed Pearson's correlations were computed for the upright and inverted faces presentations for the eye and the mouth conditions between the right FFA and the other ROIs, and results were Bonferonni corrected for multiple comparisons.

For upright faces, the time course of the FFA was not correlated with any ROIs, for either condition (see table 1). When faces were inverted, and participants were cued to the eyes, the time course in the FFA was significantly positively correlated with the IOG, LOC, FOA, IPL and IFC and negatively correlated with the SubCal cortex (*p*<0.05). When faces were inverted, and participants were cued to the mouth, the FFA was positively correlated with the same areas, plus with the SPL and left amygdala, and negatively correlated with the SubCal and mFC (*p*<0.05).

**Table 1:** Functional connectivity: Correlations between the right FFA and the other ROIs of the right hemisphere and both amygdalae.

INVERTED, THATCHERIZED MOUTH			INVERTED, THATCHERIZED EYES		
		bonferroni-corrected			bonferroni-corrected
	*r*-score	*p* value		*r*-score	*p* value
IOG	0.99	<0.001	IOG	0.97	0.002
LOC	0.99	<0.001	LOC	0.99	<0.001
FOA	0.97	0.003	FOA	0.93	0.026
SPL	0.99	<0.001	SPL	0.79	*ns*
IPL	0.97	0.002	IPL	0.97	0.003
IFC	0.99	<0.001	IFC	0.96	<0.001
mFC	−0.95	0.009	mFC	−0.87	*ns*
SubCal	−0.91	0.04	SubCal	−0.95	0.009
AMY-RH	−0.62	*ns*	AMY-RH	0.23	*ns*
AMY-LH	−0.91	0.044	AMY-LH	0.43	*ns*

UPRIGHT, THATCHERIZED MOUTH			UPRIGHT, THATCHERIZED EYES		
		bonferroni-corrected			bonferroni-corrected
	*r*-score	*p* value		*r*-score	*p* value
IOG	0.75	*ns*	IOG	0.86	*ns*
LOC	0.30	*ns*	LOC	−0.08	*ns*
FOA	−0.06	*ns*	FOA	0.20	*ns*
SPL	−0.22	*ns*	SPL	0.05	*ns*
IPL	−0.21	*ns*	IPL	−0.33	*ns*
IFC	−0.08	*ns*	IFC	−0.40	*ns*
mFC	0.39	*ns*	mFC	0.48	*ns*
SubCal	−0.08	*ns*	SubCal	−0.06	*ns*
AMY-RH	0.54	*ns*	AMY-RH	0.61	*ns*
AMY-LH	0.29	*ns*	AMY-LH	0.90	0.058

(Pearson's *r* scores and Bonferroni-corrected *p* values).

## Discussion

The goal of this study was to determine the behavioural markers and neurological correlates of discriminating thatcherized from typical faces. The behavioural markers were revealed as a large inversion effect for faces (measured relative to the inversion effect found with churches) with RTs and accuracy primarily driven by eyes than mouths. Despite eyes seemingly dominating over mouths, trials when both eyes and mouths were changed led to faster responses than trials were only the eyes changed when faces were upright. In contrast, when faces were inverted, trials where both eyes and mouths changed were not responded to any faster than trials where only eyes changed. The simplest explanation of this effect of orientation on RTs is that RTs to upright faces were subject to probability summation (redundancy gain) across eyes and mouths [15] whereas this was not the case for RTs to inverted faces. This may be a signature of configural face processing.

The neural correlates of the effects of orientation and feature-type on RTs and accuracy to discriminating upright thatcherized from typical faces were that the medial frontal and subcallosal cortex, the PreCun/Pci, the middle temporal and the parahippocampal gyri were activated more in upright than inverted face discriminations. These data are consistent with discriminating thatcherized from typical faces leading to activation of a network of areas involved in emotional processing and the making of self-referential judgements ([6], [16]–[18]).

Discriminating thatcherized from typical faces when faces were inverted activated regions of the face- and object-processing network (specifically, FFA, IOG, LOC, FOA, SPL, IFC) as well as the superior frontal cortex and the middle cingulate cortex. Furthermore, the time course of activation in this face-processing network correlated with that of the FFA. This more generalised activation in response to inverted than upright faces is consistent with previous research [4]. We suggest that decisions about grotesqueness in inverted faces are made following slavish (i.e. by multiple localised feature comparisons of restricted range) image processing, relative to upright faces. Such slavish analysis is probably based on feature analysis allowing attribution of thatcherized versus typical to be made according to certain rules (i.e. if the eyes curve upwards then faces are likely to be ‘typical’ when upright).

The impact of orientation on activation was also found in differential effect of cuing in both upright and inverted faces, across eyes and mouths. Cuing to eyes in upright faces significantly increased activation in the subcallosal cortex, compared to cuing to mouths. In contrast, cuing to eyes in inverted faces enhanced activation in the FFA and IFC, relative to cuing to mouths. These findings suggest that activation of the subcallosal cortex, the FFA and the IFC are modulated more by cuing to eyes than by cuing to mouths.

The fact that orientation influences perception of the Thatcher illusion is the essence of the illusion. What is not known is why orientation has such an effect on the perception of the illusion. One approach used in previous research that aims to answer this question has been to investigate the angle at which the shift from grotesque to normal perception occurs [3], [19], [20]. The results tend to show a quadratic/cubic function relating perception of the illusion to orientation, with a dramatic collapse in perception of the illusion around 94 to 100 degrees. Invariably these results have been interpreted as representing a qualitative shift in processing between configural and featural processing, with faces within c.100 degrees of upright being processed configurally and faces beyond this angle being processed as features. Our study only used upright and inverted stimuli and so can only be used to compare to the processing of upright and inverted faces in these previous studies. From our behavioural results, we can agree with previous studies that there is a qualitative difference between the discrimination of upright and inverted thatcherized from typical faces. However our neuroimaging data suggest that an account that explains this qualitative difference exclusively in terms of configural and featural processing may be incomplete. These data show the importance of the subcallosal and mFC activation when discriminating between upright typical and grotesque faces. In contrast, the data show discriminating between inverted faces leads to activation in a range of face processing areas, with their time courses correlated with that of the FFA. We suggest that a key element of any account of the discrimination of thatcherized from typical faces should be the activation of emotion/self-referential processing areas for upright faces. The role of configural processing in the processing of emotional faces has received some attention [21] but remains to be investigated.

There has only been one previous neuroimaging study of the Thatcher illusion [10] and there are key differences in the results of that and the present study. Almost certainly these differences reflect methodology and analytic strategy rather than inconsistency in findings. Our study differs from Rotshtein et al. [10], who used a one-back matching task to sequentially presented typical emotional faces and thatcherized versions of the same or similar faces. The authors were interested in overall activation levels in response to faces, as well as neural adaptation to repeated and different faces varying in emotional salience. The key findings related to the LOC and the AMY: thatcherization increased overall activity in the AMY and the LOC, although in the LOC this was limited to when successive stimuli were repeated and not to when successive stimuli were different. In short, thatcherization led to increased activation levels, relative to typical faces in the AMY. Importantly, the effects of thatcherization on activation were also found for inverted faces (although this comparison across upright and inverted orientations was treated as a qualitative comparison and not compared quantitatively.) In contrast, the present results are based on (1) an explicit, quantitative comparison across upright and inverted faces; (2) discrimination between simultaneously presented typical and thatcherized faces; (3) the presentation of neutral typical faces as opposed to emotional typical faces. The present data indicate that the effect of thatcherization on neural activation in response to the discrimination is equivalent across upright and inverted faces for the amygdala, but for the LOC, increased activation is observed for inverted compared to upright discriminations.

In conclusion, we have demonstrated that the discrimination of the Thatcher illusion from typical faces is associated with automatic activation of emotion/self-referential processing areas when discriminating upright faces. In addition, we have shown that discrimination amongst inverted faces, not perceived as bizarre, engage face-processing areas more than their grotesque upright counterpart.

## Materials and Methods

### Participants

After complete description to the participants of either the behavioural or the imaging study, written informed consent was obtained. Ethical approval was obtained in line with the principles of the Declaration of Helsinki. Experiment 1 was approved by the Ethics committee of the University of Southampton and Experiment 2 was approved by the Ethical Committee for Clinical Research from Lausanne Medical School and affiliated hospital. Twelve undergraduate and postgraduate students from the School of Psychology at the University of Southampton participated in the behavioural study. Participants had a mean age of 27.71 years (SD = 5.79), all had normal or corrected to normal vision, 11 were right handed, 1 was left handed, 7 were male, 5 were female. In the imaging study there were 18 healthy participants with a mean age of 27.4 (SD = 8.1). All had normal or corrected to normal vision, 15 were right handed, 3 were left handed, 13 were male and 5 were female.

### Stimuli

#### Face stimuli

Sixteen grey scale female faces were obtained from the Stirling Picture Database. These sixteen faces were used to create three sets of thatcherized faces: a set which had the eyes manipulated, a set which had the mouth manipulated and a set which had both the eyes and mouth manipulated, creating a total of 48 images. Note that a same/different control experiment run showing just pairs of eyes or mouths, presented in isolation outside of face contexts, demonstrated no significant difference in discriminability of the eyes and mouths used in the present study. See Figure 1 for an example of the stimuli. Stimuli were 9 cm in height by 8 cm subtending a visual angle of 6.86 by 6.11 degrees when viewed from a distance of 75 cm.

The blur tool was used to remove high contrast edges that are caused when manipulating images as these can act as local feature cues. Finally whole images were blurred using a one pixel Gaussian blur. Manipulated faces were paired with the non-manipulated versions of the same faces. This created three pairs of faces for each of the 16 faces. Each pair of faces was presented both upright and inverted. All manipulated faces appeared on left and right sides.

#### Church Stimuli (Behavioural task only)

Sixteen pictures of churches were obtained from sources on the internet. Churches are an appropriate control stimulus as they are familiar and have a dominant orientation. Churches were all photographed in front view. Target versions of the churches were created by manipulating the door and a window in the same way as described above. High contrast edges were removed where necessary using the blur tool and the whole image was blurred using a one pixel Gaussian blur (See Figure 1 for an example).

### Apparatus

#### Behavioural task

Two alternative forced choice (2AFC) stimuli were presented on a Viglen Genuine Intel Contender P3800 computer with a screen size of 15 inches and a refresh rate of 75 Hz. The stimuli were presented and responses (reaction times and errors) recorded using e-prime software. Responses were made using the right and left mouse keys.

#### Imaging parameters

Anatomical and functional MR images of brain activity were collected in a 3T high-speed echoplanar-imaging device (Tim Trio, Siemens, Erlangen) using a 12-channel matrix coil. Participants lay on a padded scanner couch in a dimly illuminated room and wore foam earplugs. Foam padding stabilized the head. High-resolution (1.0×1.0×1.2 mm) structural images were obtained with a magnetization-prepared rapid acquisition with gradient echoes (MP-RAGE) sequence (160 slices, 256×240 matrix, echo time (TE) = 2.98 ms; repetition time (TR) = 2300 ms; flip = 9°). Functional sessions began with an initial sagittal localizer scan, followed by autoshimming to maximize field homogeneity. Slices were automatically positioned using an on-line 3D localizer [22]. The co-registered functional acquisition (TR = 3,000 ms, 47 AC-PC slices, 3 mm thick, 3.12 mm by 3.12 mm in-plane resolution, 128 images per slice, TE = 30 ms, flip angle 90°, matrix = 64×64) lasted 384 seconds.

### Design and Procedure

#### Behavioural task

Face and church sessions were counterbalanced and completed within 24 hours. The face session had eye, mouth and uncued conditions with each condition sub-divided into four blocks of eight trials long repeated four times (i.e. 128 trials in each condition). In blocks 1 (upright faces) and 3 (inverted faces) of the eye condition, only eyes were manipulated. Participants were cued at the beginning of the block. In the mouth condition, blocks 1 (upright faces) and 3 (inverted faces) contained faces that had been manipulated only at the mouth. Participants were cued at the beginning of the block. Blocks 1 (upright faces) and 3 (inverted faces) of the uncued condition contained faces that were manipulated either at the eyes or the mouth. Participants were not cued to location of manipulated features. In Blocks 2 (upright faces) and 4 (inverted faces) of eye, mouth and uncued conditions, both eyes and mouths were manipulated. The uncued condition was repeated to allow the same number of eye and mouth trials as in the cued conditions. Condition order was counterbalanced across participants using a Latin square. The same design was used for the church session.

Blocks began with a 10000 ms cue stating “changes have been made to the eyes”, “changes have been made to the mouth” or “changes have been made to the eyes and mouth” (or a “next block” prompt in the uncued condition). Stimuli were presented until response and preceded by a 500 ms fixation cross. Participants sat approximately 75 cm from the screen in a dimly lit quiet room, were asked to keep their heads upright and to select the thatcherized face by pressing the corresponding mouse key. They were allowed a short and self-defined break between each block. Prior to starting the experiment, participants were shown examples of the four stimulus types and completed a practice session of 15 trials. Participants were told to respond as quickly but as accurately as possible.

#### Imaging task and data analysis

The design was similar to the behavioural task except that only the cued face conditions were tested. The cue preceding each block lasted for 3 seconds. Each stimulus pair was presented for 1350 ms during which participants responded. A fixation cross was then presented for 1650 ms. A face localizer alternating upright faces and objects across blocks was also obtained, for all participants, in a separate experiment [23].

FSL (FMRIB Software Library) package and techniques were used in data preparation and processing. Specifically, FSL Brain Extraction Tool (BET) was used to remove non-brain tissue [24] and fMRI data processing was performed using FEAT (FMRI Expert Analysis Tool) version 5.98 [25], [26]. Each functional run was first motion-corrected with MCFLIRT [27] and spatially smoothed with full width at half maximum of 6 mm. First-level analysis was performed using FILM (FMRIB's Improved Linear Model), which uses a nonparametric estimation of time series autocorrelation to pre-whiten each voxel's time series. High pass temporal filtering was applied to remove low frequency artefacts. Registration to standard space was achieved using FNIRT (FMRIB's nonlinear image registration tool, http://www.fmrib.ox.ac.uk/fsl/fnirt/index.html). For each of the three conditions, (discrimination between typical faces and faces modified at i) the eyes ii) the mouths, or iii) both mouths and eyes) the contrasts were between upright and inverted faces. A mixed effects GLM analysis was carried out across participants using the two stages of FLAME (FMRIB's Local Analysis of Mixed Effects). Threshold significance in the whole brain analysis was *p*<0.05 using false discovery rate (FDR) in order to correct for multiple comparisons.

*A priori* regions of interest (ROIs) were defined by anatomical or functional constraints. The anatomical constraints were specified by labels corresponding to the 25% probability Harvard-Oxford cortical atlas on a standard brain and were mapped back to each participant. The ROIs that were defined by anatomical constraints were areas involved face and emotion/social processing: SPL, IPL and IFC, medial frontal cortex (mFC), subcallosal cortex (SubCal) and the AMY. For the functional areas involved in face and object processing: FFA, IOG, medial fusiform object area (FOA) and LOC, which encompass only parts of the anatomical labels available in FSL, labels were independently created for each participant with functional data independently obtained in an experiment alternating upright faces and objects. As it is known that there is a right hemispheric dominance for face processing (e.g. [28], [29]), ROIs were selected in the right hemisphere only except for the amygdala known to be involved bilaterally in face processing.

For each anatomical and functional ROI and for each single feature condition (eye or mouth), the percentage BOLD signal change was extracted from the parameter estimate (at the participant level) for the contrast upright versus inverted faces using FSL's Featquery.

A one-sample *t*-test was conducted in order to determine whether the percent signal change values for the contrast upright vs. inverted were significantly different from zero in the eye-cued and the mouth-cued conditions. Significance level was *p*<0.01.

Differences between single feature conditions (eye or mouth) were evaluated with a paired one-tailed *t*-test and significance threshold was *p*<0.05, uncorrected for multiple comparisons. Looking at the eyes is known to increase activation in the FFA [30]. We hypothesized that cueing to the eyes would increase the time spent on the eye region and therefore used a one-tailed *t*-test to investigate whether modifications to the eyes would lead to increased activation in the FFA and other elements of the face processing network compared to modifications to the mouth.
